# The Obstacles to a Broader Application of Alkali-Activated Binders as a Sustainable Alternative—A Review

**DOI:** 10.3390/ma16083121

**Published:** 2023-04-15

**Authors:** Amina Dacić, Katalin Kopecskó, Olivér Fenyvesi, Ildiko Merta

**Affiliations:** 1 Department of Construction Materials and Technologies, Faculty of Civil Engineering, Budapest University of Technology and Economics, Műegyetem rkp. 3, 1111 Budapest, Hungary; 2 Department of Engineering Geology and Geotechnics, Faculty of Civil Engineering, Budapest University of Technology and Economics, Műegyetem rkp. 3, 1111 Budapest, Hungary; 3 Institute of Material Technology, Building Physics, and Building Ecology, Faculty of Civil Engineering, TU Wien, Karlsplatz 13, E207-2, 1040 Vienna, Austria

**Keywords:** alkali-activated binder, precursor, alkali activator, technical performance, environmental performance, economic performance

## Abstract

This paper aims to raise awareness regarding the obstacles limiting alkali-activated binders’ (AABs) application as a sustainable solution in the construction industry. Such an evaluation is essential in this industry, which has been introducing a wide range of alternatives to cement binders yet achieved limited utilisation. It has been recognised that technical, environmental, and economic performance should be investigated for the broader adoption of alternative construction materials. Based on this approach, a state-of-the-art review was conducted to identify the key factors to consider when developing AABs. It was identified that AABs’ adverse performance compared to conventional cement-based materials mainly depends on the choice of which precursors and alkali activators to employ and the regionalised practices adopted (i.e., transportation, energy sources, and data on raw materials). In light of the available literature, increasing attention to incorporating alternative alkali activators and precursors by utilising agricultural and industrial by-products and/or waste seems to be a viable option for optimising the balance between AABs’ technical, environmental, and economic performance. With regard to improving the circularity practices in this sector, employing construction and demolition waste as raw materials has been acknowledged as a feasible strategy.

## 1. Introduction

Alkali-activated binders (AABs) are prepared from solid low- or high-calcium aluminosilicate precursors in a strongly alkaline environment, thus generating hardened binders with cement-like properties. In the contemporary literature, the issue of the necessity of distinguishing between AABs and geopolymers has been widely discussed. A geopolymer is defined as a polymer created by the partial dissolution of an aluminosilicate source in a user-friendly alkaline or acidic medium to construct three-dimensional polymeric networks. On the other hand, the mechanism for the creation of AABs is similar to that of ordinary Portland cement (OPC), where, instead of calcium silicate hydrate gel, potassium or sodium aluminosilicate hydrate is available [1]. Davidovits makes a distinction even between a geopolymer cement and a binder, stating that cement refers to a binding system that hardens at room temperature (e.g., OPC), while a binder requires heat setting [2]. Although some studies tend to use these words interchangeably, the question of differentiating between these terms will not be addressed in this study. Instead, the terms used in the articles under consideration will be primarily provided as they were in the source material.

The application of AABs was introduced due to the lack of OPC availability in the post-World-War-II period [3]. Since the late 1990s, the usage of AABs has been investigated as a sustainable solution in the construction industry [4]. In the initial investigations of AABs’ environmental impacts, Davidovits stated that 1 tonne of geopolymer cement generates 0.18 tonnes of CO_2_, corresponding to an about five to six times lower value than OPC [5]. In comparison, Duxson et al. emphasised that the use of geopolymer binders can result in an 80% or even greater decrease in CO_2_ emissions compared to OPC [6]. The trend of introducing AABs as sustainable solutions but focusing mainly on their material performance was recognised while reviewing the literature. Furthermore, there was a lack of review papers tackling the topic of sustainability that considered the technical, environmental, and economic performance of the developed materials. Regarding the adoption of novel green solutions, the evaluation of solely material properties will not result in their wider usage. This paper will address possible obstacles to the broader application of AABs based on the three pillars mentioned above, focusing on its two major ingredients: a precursor and an alkali activator (AA). It will also provide a benchmark for the more mindful selection of ingredients when developing new AABs. This could hasten the steady process of the adoption of sustainable materials in the industry, which is a much-needed change considering the requirements of the codes in place worldwide [7].

In general, selecting the suitable AA depends on the precursor’s composition [3]. Two modes of AA production have been developed:

- One-part mixtures combine dry alkali powder, solid aluminosilicate raw material, and water. They are suitable for in situ applications due to their advantageous handling characteristics compared to two-part mixtures containing a viscous alkali solution. Since the usage and packaging of this type are similar to those of cement, its utilisation seems promising.
- Two-part mixtures are formed by combining an aqueous alkali solution, solid aluminosilicate raw material, and water. They have been recognised as suitable for precast applications. However, they pose a disadvantage, as they require the handling of large amounts of corrosive, hazardous, and viscous alkali solutions, which has led to the development of one-part mixtures [8].

However, precursors can be naturally occurring or industrial and agricultural by-products and/or waste. They are mainly composed of SiO_2_, Al_2_O_3_, and CaO [9]. By-products from the energy and mining industries have received much attention with respect to their use as a potential feedstock for AABs [10]. Moreover, solid waste generated throughout mining activities has been recognised as causing severe environmental pollution, and its utilization as raw material would be highly beneficial [11]. The producers of these by-products can benefit from reductions in their required storage and rehabilitation costs if the by-product is disposed of as waste. Additionally, from their sale as raw materials, some economic profit is also possible [10]. Various types of agricultural waste have also been investigated as raw materials for producing AABs [9]. On the other hand, bulk chemicals (e.g., sodium hydroxide, sodium silicate, etc.) are commonly utilised as AAs, while there have been few investigations on the use of industrial and agricultural by-products and/or wastes as alternative alkali sources [3]. In general, alkali hydroxide (ROH), non-silicate salts of weak acids (R_2_CO_3_, R_2_S, and RF), and silicic salts of R_2_O·(n)SiO_2_ are widely used, where R corresponds to an alkali metal, i.e., Na, K, or Li [4]. Table 1 presents detailed information about the types of precursors and alternative AAs available in the literature according to the best of the authors’ knowledge.

As this study will also cover AABs’ environmental and economic evaluation, widely used environmental impact assessment and cost analysis methods, namely, life cycle assessment (LCA) and life cycle costing (LCC), are introduced. LCA is a methodology used to evaluate a certain product’s environmental burden during its life cycle [12]. Based on the ISO 14,040 and ISO 14,044 standards, the methodology consists of four main steps: the definition of a goal and scope, an inventory analysis, an impact assessment, and interpretation [12,13]. While LCA is based on evaluating environmental performance, LCC is focused on cost-effectiveness. In contrast with LCA, a general standard for the application of LCC is not present [14]. However, regarding the construction industry, ISO 15686-5 provides guidelines for LCC, which concerns predicting and assessing the cost performance of the constructed assets [15]. LCC generally aims to evaluate the costs of acquiring raw materials, operation, maintenance, and disposal. It can also cover the costs of environmental impacts caused by a product (the so-called “polluter pays” principle). The incorporation of both LCA and LCC seems prudent for identifying the environmental and economic trade-offs of a particular product [14].

**Table 1:** Types of precursors and alternative alkali activators that are covered in the literature.

Used Material					Investigated Performance		
	Alkali Activator (AA)	Precursor	Description	Used Alkali-Activated Binder’s (AAB) Description	Tech.	Environ.	Eco.
AAS		[16]	Aluminium-anodising sludge (AAS) is an industrial waste produced in the anodization process of aluminium.	It was used together with rice husk ash (RHA) as a precursor, while the alkali activator (AA) consisted of sodium silicate (SS) and sodium hydroxide (SH) [16].	[16]		
ACS		[17]	Air-cooled slag (ACS) is a waste material generated by the steel-making process.	It was mixed with fly ash (FA) and activated by combining SH and SS [17].	[17]		
BL	[18,19,20]		Bayer liquor (BL) is generated during the Bayer process for aluminium production.	Concentrated BL was used as the primary activating solution while mixed with FA and silica fume (SF) as dry powders [18,19]. FA/BL geopolymers were also produced with different levels of Ca(OH)_2_ or ground granulated blast furnace slag (GGBFS) [20].	[18,19,20]		
BS		[21]	Boiler slag (BS) is a waste generated through industrial coal combustion.	It was used as a part of the precursor together with metakaolin (MK) and an activator of combined potassium hydroxide (KH) and RHA or SF [21].	[21]		
CC		[22]	Calcined clay (CC) is thermally treated clay, e.g., at 700 °C for six hours.	It was used as a part of a precursor with FA and activated by a combination of SH and SS [22].	[22]		
CKD	[23]	[23]	Cement kiln dust (CKD) is waste generated during cement manufacturing.	It was used in a one-part geopolymer with GGBFS and electric arc furnace slag (EAFS) [23].	[23]		
CSA	[24]		Cotton shell ash (CSA) is generated through the combustion of cotton shells.	It was dry-mixed with MK, thus forming a homogenous powder, and mixed with deionised water [24].	[24]		
DE	[25,26,27]		Diatomaceous earth (DE) is a sedimentary rock rich in amorphous silica.	Activating solution using DE was prepared with water and SH, while the precursor used was a fluid catalytic cracking catalyst (FCC) [25]. FA/MK binary system [26] and GGBFS [27] used as precursors were activated by DE and SH [26,27].	[25,26,27]		
DG	[28,29]		Desulphurisation gypsum (DG) is generated in coal-fired power plants when sulphur oxide is removed from exhaust flue gases (the same as the REA gypsum).	It was used as an activator in AAB together with GGBFS as a precursor [28,29].	[28,29]	[28]	[28]
DM		[30]	Dredged material (DM) is the product of dredged sediments from ports and waterways.	The AAB included a mix of FA and DM as a precursor and a combination of SH and SS as an AA [30].	[30]		
EAFS		[23]	Electric arc furnace slag (EAFS) is a waste resulting from the steel-making process.	It was used in a one-part geopolymer with GGBFS and CKD [23].	[23]		
FA		[22,28,30,31,32,33,34,35,36,37,38,39,40,41,42,43,44,45,46,47,48,49,50,51,52,53,54,55,56,57,58,59,60,61,62,63,64,65,66]	Fly ash (FA) is a fine powder that is a by-product of burning pulverized coal in power plants.	It can be used as a single precursor in AABs [31,34,36,38,39,42,56,61,62,63,64,65,66]. It was also utilised as a part of binary and ternary blended precursors with a variety of conventional and/or alternative precursors [17,22,28,30,32,33,35,37,40,41,43,44,45,46,47,50,51,52,53,54,55,57,58,59,60]. The ternary mix of waste brick powder (WBP), waste ceramic powder (WCeP), and waste concrete powder (WCP) was examined together with integrating FA in the precursor and activating it using a mix of SH and SS [48].	[22,28,30,31,32,33,34,35,36,37,38,39,40,41,42,43,44,45,46,47,48,49,50,51,52,53,54,55,56,57,58,59,60,61,62,63,64,65,66]	[28,46,47,48,59,60]	[28,60]
FCC		[25,67,68]	Fluid catalytic cracking catalyst (FCC) is a waste from the petroleum industry.	It was used as a precursor wherein AA was a combination of SH and RHA [25,67,68] or quartz [68].	[25,67,68]	[67]	
GGBFS		[17,23,27,28,29,33,35,41,42,45,46,48,49,51,52,53,54,55,56,59,69,70,71,72,73,74,75,76,77,78,79,80,81,82,83,84,85,86]	Ground granulated blast furnace slag (GGBFS) is a by-product of the iron-making process.	It was utilised to produce one-part AABs [23,28,77,85,86]. One of the studies explored sodium-carbonate-activated slag glass powder [82]. It was also used as a single source for precursor and as part of binary and ternary blended precursors with a variety of conventional and/or alternative feedstocks [17,27,28,29,33,35,41,42,45,46,49,51,52,53,54,55,56,59,67,69,70,72,73,75,76,78,81,83,84]. Additionally, mixed precursors containing different ratios of rice husk (RH), RHA, MK, palm oil fuel ash (POFA), and GGBFS were investigated [79]. Moreover, the ternary mix of WBP, WCeP, and WCP was examined together with integrating GGBFS in the precursor [48].	[17,23,27,28,29,33,35,41,42,45,46,48,49,51,52,53,54,55,56,59,69,70,71,72,73,74,75,76,77,78,79,80,81,82,83,84,86]	[28,46,48,59,81,85]	[28,85]
K		[73]	Kaolin (K) is an aluminosilicate crystalline mineral that is transformed into MK when heat-treated.	As a precursor, it was investigated while being activated by KH [87].	[87]		
LS		[88]	Ladle slag (LS) is derived from the steel-refining process via arc electric furnace technology.	It was either used alone as a precursor or together with MK, where AA was mixed with SH and SS [88].	[88]		
MK		[21,42,43,44,45,48,57,63,73,79,81,85,87,88,89,90,91,92,93,94,95,96,97,98,99,100,101,102]	Metakaolin (MK) is a product of calcinating kaolin.	It was used as a single precursor [21,42,63,81,85,87,89,90,91,93,94,95,100]. In the case of binary [21,43,44,45,57,73,88,92,95,96,97,98,99,101,102] and ternary [101,102] precursors, it was used together with a variety of conventional and/or alternative sources [21,43,44,45,57,73,88,92,95,96,97,98,99,101]. Mixed precursors containing different ratios of RH, RHA, MK, POFA, and GGBFS were also investigated [79]. Moreover, the ternary mix of WBP, WCeP, and WCP was examined together with integrating MK in the precursor [48].	[21,42,43,44,45,48,57,63,73,79,81,87,88,89,90,91,92,93,94,95,96,97,98,99,100,101,102]	[81,85,101,102]	[85]
MWIA		[31,40]	Municipal waste incineration ash (MWIA) is waste obtained after the incineration of municipal waste.	It was utilised as a single precursor [31] but also in ternary blended precursors, including RHA and FA [40].	[31,40]		
MWW		[103]	Mineral wool waste (MWW) is generated while manufacturing mineral wool (e.g., stone and glass wool) before adding organic resins or other additives.	It was investigated as a precursor, which was activated by SS, SH, sodium aluminate, or sodium carbonate solutions [103].	[103]		
NP		[69,104,105,106,107,108]	Natural pozzolan (NP) is a powder form of volcanic rock mainly containing amorphous silica and alumina.	It was utilised as a single precursor [104,105,106,107] but also as in binary blended precursor with GGBFS [69] and MK [108].	[69,104,105,106,107,108]		
OBA	[109,110]		Olive biomass ash (OBA) results from the combustion of olive stone [109] or olive oil industrial waste, e.g., pruning debris, leaves, and second-press olive dregs [110].	It was used mixed with water as an activator in FA- [110] and GGBFS-based AABs [109,110].	[109,110]		
ONS	[59]		Olivine nano-silica (ONS) is produced by dissolving olivine in acid at low temperatures.	It was used as an AA with SH for GGBFS/FA-based AABs [59].	[59]	[59]	
POFA		[79,84,97]	Palm oil fuel ash (POFA) is a palm oil production waste.	In the case of binary blended precursor, it was used with MK [92] and GGBFS [84]. Mixed precursors containing different ratios of RH, RHA, MK, POFA, and GGBFS were also investigated using SH as an AA [79].	[79,84,97]		
PD		[77]	Phyllite dust (PD) is a powder waste generated while manufacturing roof tiles.	It was utilised with SS, SF, and GGBFS to produce one-part GGBFS-based AABs [77].	[77]		
PS	[76]		Paper sludge (PS) is a waste product from the paper industry.	It was utilised as the CaCO_3_ source in combination with SH to be used as AA with GGBFS as a precursor [76].	[76]		
RH		[79]	Rice husk (RH) is agricultural waste generated through rice production.	It was used as a part of mixed precursors containing different ratios of RHA, MK, POFA, and GGBFS [79].	[79]		
RHA	[21,25,26,41,67,68,111,112,113,114,115]	[16,32,37,40,57,60,79,116,117]	Rice husk ash (RHA) is generated through the combustion of rice husks.	The activator was produced using a mix of SH and RHA [16,21,25,26,41,67,68,111,112,113,114,115]. When RHA was used as a precursor, it was a component of the binary and ternary systems with a variety of conventional and/or alternative feedstocks [32,37,40,57,60,116]. Additionally, mixed precursors containing different ratios of RH, RHA, MK, POFA, and GGBFS were evaluated [79]. On the other hand, a one-part geopolymer synthesized by mixing RHA and solid sodium aluminate with subsequently added water was studied [117].	[16,21,25,26,32,37,40,41,57,60,67,68,79,111,112,113,114,115,116,117]	[60,67,114,118]	[60,114]
RM	[28,29,119]	[28,58,72,80,98,99,120,121,122,123,124]	Red mud (RM) is an alkaline sludge resulting from the Bayer process, which is the production of alumina from bauxite.	It was used as an ingredient of AA for GGBFS [28,29] and FA-based AABs [119]. Additionally, it was utilised in binary and ternary precursors with a variety of conventional and/or alternative feedstocks [28,58,72,80,98,99,120,122,123,124]. Moreover, a one-part geopolymer was investigated by calcinating RM with SH pellets [121,124].	[28,29,58,72,80,98,99,119,120,121,122,123,124]	[28]	[28]
RMS		[80]	Refuse mudstone (RMS) is a by-product of blasting operations in open-cut mining that is usually located at the top of a coal seam.	It was used as a part of a ternary precursor with RM and GGBFS while utilising SS as an AA [80].	[80]		
RPP		[35]	Rock phosphate powder (RPP) is a pulverised form of naturally sourced rock phosphates.	A ternary precursor concerning the addition of GGBFS and FA activated by SH was studied [35].	[35]		
SA	[115,125]	[78]	Sugarcane ash (SA) is a residue from sugar production.	It was part of AA with SH for FA- [115] or GGBFS- [125] based AABs. As a precursor, it was blended with GGBFS [78].	[78,115,125]		
SF	[28,76,126]	[77,98,124]	Silica fume (SF) is a by-product of smelting for the manufacture of silicon and ferrosilicon alloys.	A combination of SF and SH was used as an activator for GGBFS-based AAB [28,76,126]. It was mixed with MK [97] or RM [123] as a precursor. It was also utilised as an ingredient of one-part GGBFS-based AABs [77].	[28,76,77,98,124,126]	[28]	[28]
SiMnF		[72,127]	Silicomanganese fume (SiMnF) is an industrial by-product resulting from the production of silicomanganese alloy via carbothermic reduction.	It was used as a precursor activated by a mixture of SH and SS [127]. Additionally, it was utilised as a part of the binary blended precursor with GGBFS [72].	[72,127]		
SW	[128]		Soda waste (SW) is a by-product of sodium carbonate production that is formed through the Solvay process.	It was used for the alkali activation of GGBFS [128].	[128]		
WBA	[129]		Wood biomass ash (WBA) results from the combustion of wood biomass.	It was utilised with class F pulverised fuel ash as a precursor [129].	[129]		
WBP		[27,46,47,48,81,96,102,127,130,131,132,133]	Waste brick powder (WBP) is generated from brick construction and demolition waste.	It was used as a single precursor [81,102,127,130,131,132,133] or as part of binary blended precursor with a variety of conventional and/or alternative feedstocks [27,47,96]. As a part of the ternary precursor, it was mixed with FA and GGBFS [46] as well as waste glass powder (WGP) and MK [102]. Additionally, a ternary mix of WBP, WCeP, and WCP was examined together with FA in the precursor [48].	[27,46,47,48,81,96,102,127,130,131,132,133]	[46,47,48,102,127,130]	
WCP		[48,134]	Waste concrete powder (WCP) is a by-product of concrete construction and demolition waste.	It was used as a single precursor [134] and a part of a ternary mix with WBP and WCeP integrating FA in the precursor [48].	[48,134]	[48]	
WCeP		[28,48,81,101,135]	Waste ceramic powder (WCeP) is produced from ceramic tiles discarded as construction and demolition waste.	It was utilised as a single source for precursor [81,101] and as part of binary [28,135] and ternary blended precursors [101]. A ternary mix with WBP and WCP was examined together with integrating FA in the precursor [48].	[28,48,81,101,135]	[28,48,101]	
WGP	[101,131,136,137,138]	[49,50,52,82,83,92,101,102,122]	Waste glass powder (WGP) is generated from glass, an abundant waste product since a large portion is not recycled, e.g., due to its grain size.	As an activator, it has been synthesised with SH [101,102,131,136,137,138]. As a precursor, it was employed alone or [50] as a partial replacement of MK [92,101,102] and GGBFS [83]. Furthermore, it was evaluated as a part of a ternary precursor [49,52,101,102,122]. One of the studies also explored sodium-carbonate-activated slag glass powder [82].	[49,50,52,82,83,92,101,102,122,131,136,137,138]	[101,102]	[138]

## 2. Obstacles Related to Technical Performance

The factors influencing the synthesis of AABs are the composition, fineness, quantity, and source of the precursor material; the type and composition of the AA employed; the quantity of free water used; and the type and duration of curing. These aspects affect both the fresh and hardened properties of AABs as well as their long-term performance. Various studies have investigated the effect of the chemical composition of AABs’ ingredients on their material properties. For instance, the mechanical properties of metakaolin (MK)-based geopolymers have been investigated with respect to the effect of Si/Al, Al/M (M is an alkali cation), and H_2_O/Na_2_O. In a study conducted by Lahoti et al., the Si/Al ratio was the most significant factor influencing compressive strength, followed by the Al/Na ratio [139]. On the other hand, the calcium content of ground-granulated-blast-furnace-slag (GGBFS)-based AABs seems to influence its mechanical properties, wherein excessive calcium content can confer an adverse effect [140]. Furthermore, control over the fresh properties is difficult, which is especially important because of the scarce availability of suitable admixtures. Notably, an excessive amount of water present in the mix of AABs decreases their strength because it is not essential in chemical reactions, unlike cement hydration [9]. It is mostly physically bonded and tends to increase the porosity of alkali-activated material (AAM). It may also negatively influence mechanical and durability properties [141].

One of the challenges in the broader application of AABs is the curing method employed. AABs based on glassy aluminosilicates (e.g., fly ash) typically require elevated curing temperatures for stable hydrate reaction and to gain strength [4]. Heat curing is performed in a moderate temperature range from 20 to 80 °C [142]. However, the advantageous material property of AABs is their thermal stability, which can be beneficial when the construction materials used must be thermally stable [133]. Additional issues are based on a scarcity of data, an incomplete understanding of the mix’s design, and limited information on long-term performance. Finally, the scarcity of the standards, specifications, and regulations for the production and application of AABs also hinders their broader utilisation [9]. This section will discuss the role of the type of precursor and AA used in AABs’ technical performance.

### 2.1. Precursors

**Figure 1:**
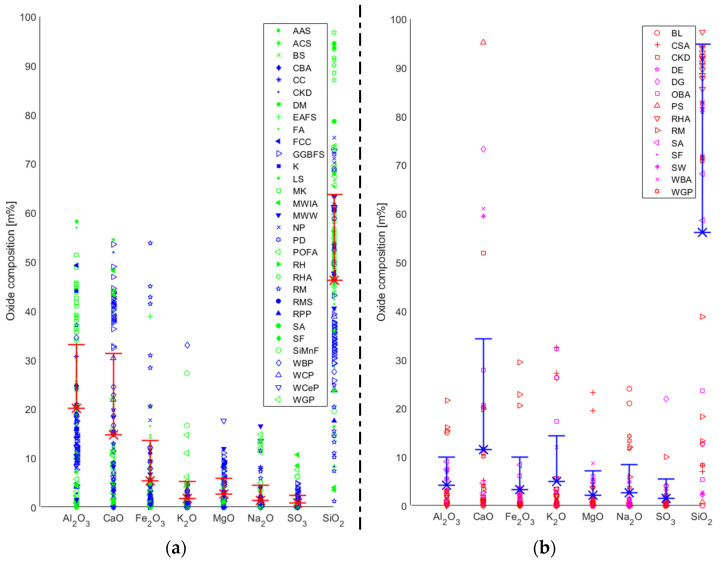
Chemical composition of (**a**) precursors and (**b**) alternative alkali activators.

As mentioned above, one of the largest concerns in this field is insufficient uniformity in the composition of the precursor materials and hence its impact on the resulting AABs [9]. It has been recognised that AABs are highly sensitive to the chemical composition of the precursor, which varies based on the source material (Figure 1a). One of the most important factors for producing a stable AAB is the use of highly amorphous precursors possessing adequate reactive glassy content. Additionally, its water demand should be relatively low, and it should release easily [140].

**Figure 2:**
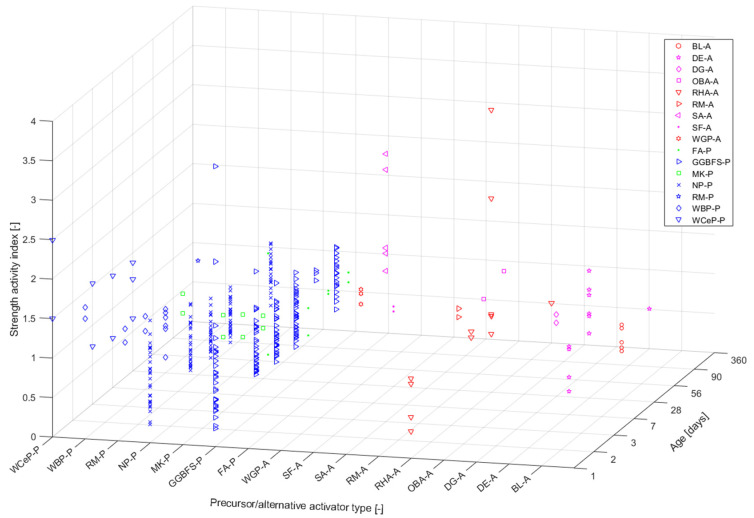
Strength ratio results for alkali-activated materials.

Figure 2 shows the strength ratio results representing a ratio of the compressive strength of the generated AAM and OPC-based material with respect to the specific precursor type used (i.e., cement paste, mortar, or concrete). The colour- and symbol-coding follows the scheme used in Figure 1a for the respective precursors. Each precursor type was assigned a code, X-P, where X corresponds to an abbreviation of the respective raw material (see Table 1) and P stands for precursor (another code, X-A, will be discussed in the next section). The data presented in Figure 2 demonstrate results obtained for mortar [28,81], concrete [55,69], and paste specimens [130]. Only papers referencing an OPC-based material are presented. For the most part, a combination of sodium silicate (SS) and sodium hydroxide (SH) was used as an activator, with varied concentrations and combinations (with some exceptions regarding the use of alternative AAs [28]). Few of the considered studies investigated AAMs’ performance using heat curing [55,81]. These discrepancies are not comprehensively discussed herein since solely the possible variability of the final compressive strength results based on the type of precursor used is evaluated in this study. The wide range of strength ratio results shows the AAMs’ mechanical-performance-related variability based on the type of precursor used. The strength ratio values ranged from about 0.2 to 3.6, where the lowest value (about 0.2) corresponded to a mix made with waste brick powder (WBP) as a part of the precursor, while the highest value (about 3.6) was associated with incorporating GGBFS into the precursor.

The reactivity of the precursors used to date cannot be simply based on their broad typologies. Silica and alumina from the source materials are among the most influential factors with respect to the material properties of AAB. Another oxide that significantly contributes to the reaction kinetics of AAB materials is that formed with calcium. It has been demonstrated that the workability of AAMs is reduced when a low-calcium precursor (e.g., fly ash) is partially/fully replaced by a high-calcium precursor (e.g., GGBFS). In general, the increase in the amount of calcium increases the rate of hydration (which is the opposite chemical process to polymerisation) while also consuming water. Due to these two effects, the viscosity can be increased, while the setting time can be decreased of the resulting AAM. Furthermore, Xie et al. [141] defined a threshold value for the content of CaO in AABs of 8%. When this value is surpassed, the effect of hydrates from the reactions involving CaO on the compressive strength of alkali-activated concrete (AAC) is more pronounced. In contrast, when the content of CaO is lower than 8%, the mechanical strength of the AAB is mainly contributed by the geopolymeric products (from alumina, silica and iron (III) oxide) [141].

Moreover, heat curing is usually applied to low-calcium systems because of the slow polymerisation process, which is also one of the major disadvantages of utilising this kind of AAC cast in situ. However, when GGBFS is considered, air-dry curing can be applied [4]. Moreover, mixing low-calcium fly ash (FA) and high-calcium GGBFS has been recognized as a solution that can result in air-dry curing due to the increased composition of calcium in the precursor. Finally, iron (III) oxide has been shown to inhibit C-A-S-H formation in hydrated high-calcium AAMs. The material properties of low-calcium AABs can be improved by replacing Al^3+^ with Fe^3+^ in the product’s octahedral sites [141]. Hence, four oxides, namely, CaO, SiO_2_, Al_2_O_3_, and Fe_2_O_3_, can be recognised as the most influential for the material properties of AABs when the precursor is considered.

The database generated in this study (based on the literature) showed that the significant oxides in precursors are SiO_2_, Al_2_O_3,_ CaO, and Fe_2_O_3,_ with mean percentages of 46.2%, 20.1%, 14.7%, and 5.4%, respectively (Figure 1a). Based on all the data points, the standard deviations (SDs) for silica and alumina are 17.5% and 13.0%, respectively. The precursors with the most data points, and hence the most widely used ones, are FA, GGFBS, and MK. With regard to FA, 58 data points were gathered, and the mean value and SD of the presence (in m%) of silica was determined to be about 51.3% and 8.8%. On the other hand, the mean alumina value was 25.3%, for which the SD was 6.9%. While the number of data points for GGFBS is 39, the mean values for silica and alumina are 33.6% and 11.7%, respectively. At the same time, the SD is equal to 3.7% for silica and 2.4% for alumina. Lastly, 26 data points for MK were collected; the mean value and SD for silica are 53.2% and 4.8%. The mean of alumina is 40.6%, for which the SD is 5.5%.

The variability of the chemical composition of the precursors is most visible in the case of alternative precursors (i.e., alternatives to widely used precursors such as FA, GGBFS, and MK). Since there is a lack of wide-ranging investigations based on different sources, the conclusions in these studies would be limited to the specific precursor type used and the source from which it was generated. The studies in the literature have used various methods to predict the material behaviour of AABs based on, among other factors, the chemical composition of precursors. However, the shortcomings of these approaches have been identified, which were either based on the use of a limited dataset or the exclusion of certain factors proven to be essential for determining the material behaviour of AABs (e.g., the curing method employed). It seems essential to generate databases and methods that can comprehensively cover all the factors and possible variables in future studies. This is especially the case for alternative precursors, for which the precursor’s chemical composition variability can be high based on the source. This is demonstrated in Table 2, where alternative precursors with the highest quantities of collected data points are presented: red mud (RM) [28,58,72,80,98,99,120,121,122,123], rice husk ash (RHA) [16,32,37,40,57,79,116,117], WBP [27,46,47,81,96,102,127,130,131,132,133], and waste glass powder (WGP) [49,50,52,82,92,101,102,122]. It can be seen that both the types of oxides that are the most abundant and their SD values differ significantly. However, RHA seems to be the alternative precursor with the most stable chemical composition with a substantially high presence of silica. Therefore, aside from being investigated as a precursor, its application as an AA has also been widely studied.

### 2.2. Alkali Activators

The AAs suitable for AABs could be any sufficiently water-soluble compound whose use results in a high pH and that is able to provide alkali/alkali earth cations (e.g., alkali-hydroxides/sulfates/carbonates/silicates) [3]. The adverse effects of the high quantity of AAs on the cost of production, strength gain, and performance in aggressive environmental conditions has been acknowledged [9]. Some of the most used AAs are SH and SS. Generally, SS results in a more compact and denser material with higher mechanical strength than SH. It also offers greater advantages regarding handling and usage compared to aqueous activators [8]. In AABs’ systems, alkali hydroxides (e.g., SH) provide OH^−^, and, based on the interdiffusion and ion exchange of solutes and aluminosilicate network modifier cations (e.g., Ca^2+^) of the precursors, the dissolution of the precursor is promoted. The dissolution of most of the precursors increases as a function of an increasing alkali hydroxide concentration. However, a considerable concentration of the alkali hydroxide in AABs can result in efflorescence problems. On the other hand, when silicate-based activators are used, the precursors’ activation mechanism is similar to hydroxide-based activators with additional soluble silicate available. Moreover, carbonates, as activators, could aid CO_2_ binding in the presence of calcium in the system. Adding alkali sulphates as activators can be a viable solution for durability enhancement, wherein chloride binding properties can be introduced. Nevertheless, alkali carbonates and sulphates are unsuitable for low-calcium precursors (e.g., MK) due to the relatively low pH value introduced in the mix. In comparison, alkali sulphates result in a substantially lower heat of hydration than most other activators, which could be beneficial for application to AABs. Finally, aluminate-based activators constitute another type of AA, which offer the benefit of dissolved alumina [3].

However, dual AAs of SH and SS have been recognised as highly efficient for use in AABs containing both low and high calcium content. In such a system, SH is the main activator providing hydroxide anions (OH^−^), whereas SS acts as an auxiliary activator providing soluble silica. Moreover, the effect of AAs on the final and initial setting times has been recognised, where at a higher dosage of SS, the increased SS/SH ratio causes an accelerated rate of dissolution of the precursor, thereby altering reaction kinetics and the condensation process. This leads to fast initial and final setting times. However, the SS/SH ratio or the concentration of SH causing an increase in the overall liquid content to binder ratio substantially delays setting. The effect of the usage of other types of activators on the material properties of AAMs is discussed extensively in a study by Xie et al. [141], where the reader can find more detailed information. The findings of Xie et al. (which did not cover studies on alternative activators) suggest that the contribution of a structure-forming element to the strength of concrete depends on the solubility of the auxiliary and basicity of the main activator [141].

Since environmental issues are a significant concern in the industry and there are widely available industrial and agricultural waste streams with no market value, alternative solutions to widely used primary AAs have been extensively investigated. Such approaches are especially viable for AA because, for example, silica can be extracted from glass waste such as RHA, silica fume (SF), and many other biomass ashes and combined with alkali hydroxides through hydrothermal or thermochemical methods, resulting in the production of SS [3]. In Figure 2, the strength ratio results of AAMs containing alternative activators are presented (A is used as the designation for the used activator, e.g., an activator containing SF is designated as SF-A). The references used for calculating the strength ratios were mixes based on the conventional AA presented in the study, e.g., a mixture of SH and SS. The results ranged from about 0.3 to 3.6, with the lowest value (0.3) corresponding to a mix containing diatomaceous earth (DE) as an AA, and the value of 3.6 corresponding to RHA.

In Figure 1b, the chemical compositions of various types of industrial and agricultural waste used to generate AAs are presented. We can conclude that the principal oxides in the alternative AAs considered in this study are SiO_2_, CaO, K_2_O, and Al_2_O_3_ with average mass content values of 56.2%, 11.5%, 4.9%, and 4.2%, respectively. However, the use of alternative sources for AA has not been as extensively studied as for precursors. The highest number of data points was gathered for RHA [21,25,26,41,67,68,111,112,113,114,115]. This was expected since, in the previous section (where alternative precursors were presented in Table 2), RHA was recognized as a source with the most constant chemical composition and a high amount of silica (higher than 90%). Its pH is near neutral, so it is unsuitable for replacing SH-based AAs. RHA’s annual production is estimated at around 40 Mt [3]. On the other hand, RHA is not a type of waste that is abundantly available in all regions. The countries with an estimated availability of RHA of more than one million tons per annum are China, India, Indonesia, Bangladesh, Vietnam, Thailand, and Myanmar [143]. Hence, focusing on widely available local resources could be a more efficient solution for the broader utilisation of such AA alternatives. Accordingly, economic and environmental benefits would also be comparably higher.

## 3. Obstacles Related to Environmental Performance

**Figure 3:**
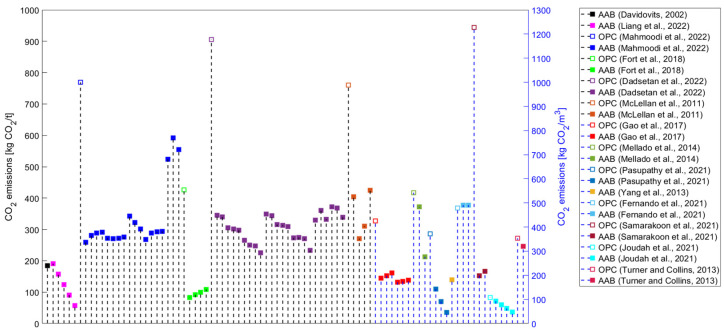
CO_2_ emissions results for alkali-activated materials from the literature [4,5,10,46,48,59,60,67,101,102,127,130,144,145,146].

**Figure 4:**
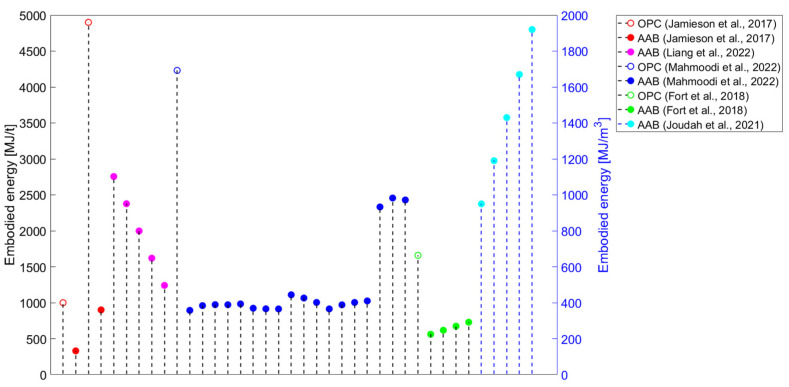
Embodied energy results for alkali-activated materials from the literature [19,46,48,130,145].

Although studies identifying AABs as constituting a sustainable alternative to OPC have been published since the beginning of this century, studies that systematically approach the environmental performance of AAMs are still scarce. If only greenhouse (GHG) emissions (i.e., global warming potential—GWP) are evaluated in environmental impact assessments, the production of AAMs could lead to lower values than those contributed by conventional materials. However, other than GWP, various environmental impact categories should be assessed, such as ozone layer depletion (ODP), acidification potential (AP), human toxicity (HTP), etc. Even in one of the first studies tackling the environmental performance of AABs, Davidovits not only touched upon CO_2_ emissions but also the heavy metal waste encapsulation possibilities of geopolymer cement as an environmentally driven application [5]. The results could be surprising if a comprehensive evaluation is performed, wherein it can be observed that AAMs are projected to be less sustainable than widely anticipated [3]. Most studies have focused on estimating AAMs’ GHG emissions (Figure 3) or embodied energy (Figure 4). Even if only the evaluation of these two environmental impact categories is considered, a comparison of the results of the studies is complex because there are discrepancies in the methods used (Table 3).

Some of the most critical steps in LCA are defining functional units and system boundaries. A functional unit is defined as the quantified performance of a product system for use as a reference unit. At the same time, a system boundary is a set of criteria specifying the unit processes forming part of a product system [12]. The definition of these two steps is essential for an LCA of a construction material because when compared to other solutions, care should be taken as to how this comparison would be conducted. For example, the material with the lowest environmental impact per unit volume or mass is not always the one with the lowest environmental impact when used in a structure throughout its service life [149]. Based on the data gathered (Table 3), functional units were mainly defined based on mass (i.e., t) or volume (i.e., m^3^). On the other hand, system boundaries differed significantly. Some of the studies considered the cradle-to-gate approach, which would include raw material production and the mixing of the constituents. However, even the definition of the unit processes covered by such a system boundary varied, with some of the investigations excluding/including either data on mixing ingredients and transportation or the heat-curing methods required for AAMs. When it comes to heat curing, numerous studies have been based on ambient temperature, and hence environmental impacts connected to curing were not considered [8,24,43,46,59,67,96,102,130,145]. However, various studies did not cover heat curing, although it was required for the production of a specific AAB [10,48,127].

Ultimately, it is debatable whether the cradle-to-gate approach, which should focus solely on producing certain products, should include heat curing (i.e., not installation on site). On the other hand, some of the studies included transportation data in their evaluation and even extended the system boundary to cradle-to-preconstruction [4]/site [147]/end of construction [60]. By neglecting transportation data, conclusions that are not representative of the real situation could be drawn because the availability of most of the raw materials could significantly differ between conventionally available ones (e.g., OPC) and their alternatives (e.g., GGBFS+SS system). If we compare AAMs and OPC-based products, especially if some of the raw materials for AABs originate from a specific waste stream, transportation distances play a major role. These aspects hinder the comparison of the results from various studies (e.g., the developed goods). However, Figure 3 and Figure 4 show mainly reduced embodied energy and GHG emissions when an OPC-based material is assessed as a reference in a given study and compared with AAMs.

Moreover, adopting well-developed and well-known LCA methods such as CML and ReCiPe could provide a more comprehensive environmental impact assessment of AAMs. Based on the data in Table 3, only five studies considered the use of one of these methods. This implies that even the studies that did not consider GWP as the only environmental impact category mainly assessed embodied energy as an additional category. Overall, the constituents of AABs (namely, an AA and a precursor) play a significant role in the final environmental burden of AAMs.

### 3.1. Precursors

When OPC concrete was compared with AAC using GGBFS and FA, the obtained levels of carbon emissions were two times lower [59]. The use of MK as a precursor was compared to secondary raw materials (i.e., FA and GGBFS), which were associated with AABs’ more favourable environmental impacts [142]. Ameri et al. showed that using various types of precursors, namely, GGBFS, MK, waste ceramic powder (WCeP), and WBP, for AAM could lead to an up to 50% reduction in CO_2_ emissions and a 25% decrease in energy consumption compared to cement mortar [81]. Compared to merely the binary precursor of GGBFS and FA, the ternary precursor of GGBFS, FA, and WBP reduced GHG emissions and energy consumption by roughly 70% and 55%, respectively. [46]. Joudah et al. [145] investigated the energy usage and CO_2_ emissions of AAMs developed from FA, GGBFS, and WCeP. While the crushing, sieving, and grinding of WCeP were covered, the crushing of GGBFS was excluded, and no laboratory treatment was conducted with respect to FA. The results showed that GGBFS had the least favourable results in all categories, while FA’s values were the lowest. This can be explained by the higher electricity and diesel consumption required to produce GGBFS. About four- and six-times higher values were obtained for WCeP compared to FA concerning CO_2_ emissions and energy consumption, respectively. Ternary binders exhibited lower CO_2_ emissions values than OPC for all mortar mixtures, attaining over 75% reductions in this parameter. With regard to energy consumption, all AAM mixtures yielded more beneficial results, attaining a decrease in this value by 60%. Overall, it was recognised that AAMs were the more sustainable solutions, and all the indicators declined as the substitution rate of GGBFS with FA increased [145]. Additionally, a reduction of 64% [101]–75% [102] in GHG emissions compared to an OPC binder was attained when a ternary precursor containing WGP, MK, and WCeP was utilised [101].

Moreover, in a study that fully utilised construction-and-demolition-sourced waste as WBP, WCeP, and waste concrete powder (WCP) in a ternary precursor, a decrease of about 86% in embodied energy and 76% in carbon dioxide compared to an OPC binder was shown [48]. Even the utilisation of solely WBP as a precursor resulted in a reduction of consumed energy by 63 [130]–85% [47] and GHG emissions by 78 [47]–81% [130] for a mixture of alkali-activated paste with the best mechanical properties compared to a cement one. Foam concrete investigation using WBP as a precursor has shown that GHG emissions can be reduced by up to 88%. However, it was emphasised that when choosing a suitable product, one should also consider the intended application with respect to the required mechanical and thermal properties [127]. In a study where WGP was used as both the partial activator and precursor in the paste, a decrease of 82% in CO_2_ emissions compared to an OPC binder was obtained (see Section 3.2 for a detailed description of the pastes’ mixtures) [146]. The studies showing the environmental benefits of utilising feedstocks from construction and demolition waste for precursors demonstrate that such practices could greatly improve the more comprehensive sustainable approaches in the construction industry by focusing on circularity.

**Figure 5:**
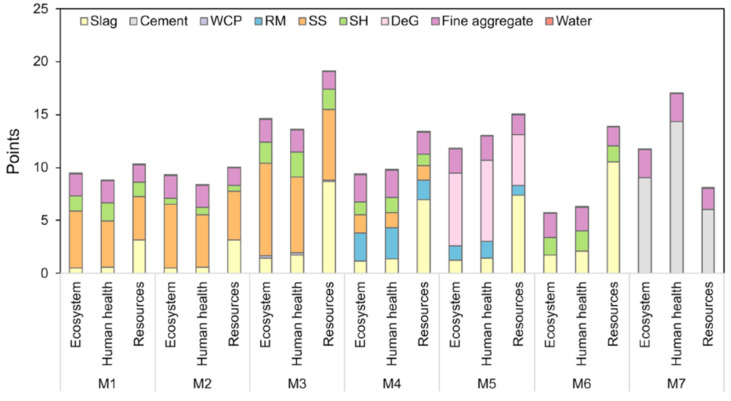
The endpoint results of the environmental assessment for the following AAMs: M1 (precursor—FA + GGBFS; AA—SS + SH; and activator modulus—1.00), M2 (precursor—FA + GGBFS; AA—SS + SH; and activator modulus—1.40), M3 (precursor—WCeP + GGBFS; AA—SS + SH; and activator modulus—1.02), M4 (precursor—RM + GGBFS; AA—SS + SH; and activator modulus—1.00), M5 (precursor—GGBFS and AA—RM + DG), M6 (precursor—GGBFS and AA—SF + SH), and M7 as OPC mortar [28].

Ramagiri and Kar [28] extensively studied AAMs by replacing conventional precursors (FA and GGBFS) with WCeP and RM. Seven different mixtures were investigated, one of which was OPC mortar (M7), while the other six (M1–M6) were AAMs, as described in Figure 5. Regarding the first three mixtures (M1, M2, and M3), the highest environmental impact was associated with M3, which was attributed to an increase in the dosage of activators required to obtain the same compressive strength. ReCiPe endpoint scoring was used, where M6 showed the most favourable results for ecosystem quality while M3 showed those that were least favourable. In addition, when human health was considered, M6 achieved the lowest environmental impact, while OPC mortar had the highest. Based on resource depletion, M2 and OPC mortar showed the best results, while M3 presented the worst. The authors concluded that the residues with minimal pre-treatment seemed more feasible [28]. Another study utilising RHA from agricultural waste as part of the precursor together with FA was investigated. The contribution of FA and RHA to the GHG emissions of AACs (precursor mixtures: first–FA; second–FA/RHA, with a ratio equal to 90/10) was recognised as insignificant, i.e., contributing about 1% according to Fernando et al. [60]. When the normalised results were evaluated, FA and RHA accounted for only 1–3% of the investigated impact categories. Adding RHA to the precursor decreased GHG emissions by about 1 kg CO_2_-eq/m^3^. The utilisation of FA and RHA can be beneficial for addressing freshwater ecotoxicity (FWE) and marine water ecotoxicity (MWE) by avoiding the disposal of waste in dumpsites, rivers, and storage lagoons [60]. Hence, a comprehensive approach to the effect of the utilisation of industrial and agricultural waste as a (part of) precursor should be adopted to evaluate its benefits.

However, based on the type of precursor used (i.e., low-calcium precursors), heat curing plays a significant role in LCA. Turner and Collins [144] demonstrated that the contribution of curing to the production of a geopolymer, for which the precursor was FA (a temperature of 60–80 °C for 24 h was implemented), is more than 40 times that of OPC concrete. Heat curing contributed about 12% to the total CO_2_ emissions associated with geopolymer concrete production [144]. Sandanayake et al. [147] also found that for the GHG emissions of FA geopolymers, one of the major contributors is heat curing. They conducted a building-level case study, wherein the optimisation of the prefabricated and on-site concrete amounts was investigated to minimise GHG emissions. This strategy can produce favourable results because AACs can be used for the prefabricated concrete portion of a project due to the requirement for thermal curing. [147]. On the other hand, in the study by Fernando et al. [60], the contribution of heat curing to GHG emissions was about 9% for the two AAC types studied, where only FA as a precursor was used for the first type and for the other a mixture of FA (90%) and RHA (10%) was employed. Optimising the heat-curing process while maintaining strength requirements was recognised as a path for reducing the environmental burden of AACs [60].

**Figure 6:**
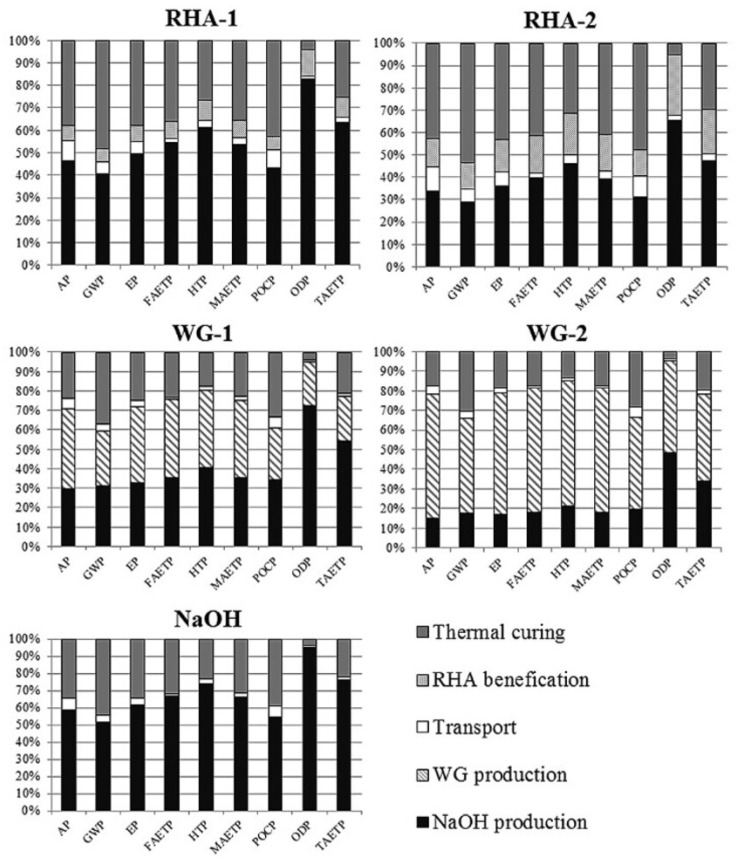
Contribution of heat curing to the LCA results of the binders where precursor was CKS and AAs were SH, RHA-1/WGP-1, and RHA-2/WGP-2, representing the combination of RHA/WGP, SH, and water with different SiO_2_/Al_2_O_3_ molar ratios of 3.5 and 4.05, respectively [118].

Weil et al. [142] conducted a comparative LCA between geopolymer concrete with a GGBFS/FA ratio of 80/20 (for which heat curing was unnecessary, i.e., air-dry curing was applied) and OPC. Three environmental impact categories were examined: AD, GWP, and cumulative energy demand (CED). The AD and CED results were similar between the two concretes, but for GWP, the geopolymer had an about 70% lower value. They also concluded that heat curing should be reduced to a minimum in the case of geopolymer production. Using waste heat from other processes to decrease environmental impacts was considered to be one of the solutions [142]. Moreover, the LCA conducted by Passuello et al. [118] covered the use of cement kiln dust (CKD) as a precursor for generating geopolymer paste (see Section 3.2 for the AAs used). The impact categories evaluated using the CML method were eutrophication potential (EP), FWE, GWP, HTP, MWE, photochemical ozone creation potential (POCP), ozone depletion (ODP), and terrestrial ecotoxicity (TAETP) (Figure 6). The results indicated that the thermal curing of geopolymer pastes substantially contributes to all of the impact categories but ODP. They also concluded that these impacts could be majorly decreased by avoiding the application of thermal curing. In the case of GWP, this reduction can be as much as 50% [118]. However, focusing on generating AABs using construction and demolition waste as a single/partial precursor by curing in an ambient temperature could lead to major environmental benefits and the improvement of the circularity of this industry, which was demonstrated in [24,43,46,95,96,130,145].

### 3.2. Alkali Activators

One of the first LCAs of the AABs was performed by Weil et al. in 2009. They emphasised that the application of SS and SH should be reduced or replaced by more eco-friendly activators [142]. Around 60 Mt of SH is produced annually, while the amount of SS is around 1/10th of this value [150]. SS has been identified as one of the most significant contributors to the environmental impacts of AAMs, which is mainly due to the high level of energy consumption related to its production [8]. In the literature, conventional AAs were recognised as having a significant impact on AAMs’ GHG emissions [4,10,28,47,81,101,102,127,130,144,147]. It is estimated that 1.1 kg and 1.2 kg of CO_2_ are emitted per 1 kg of SH and SS production, respectively [3].

**Figure 7:**
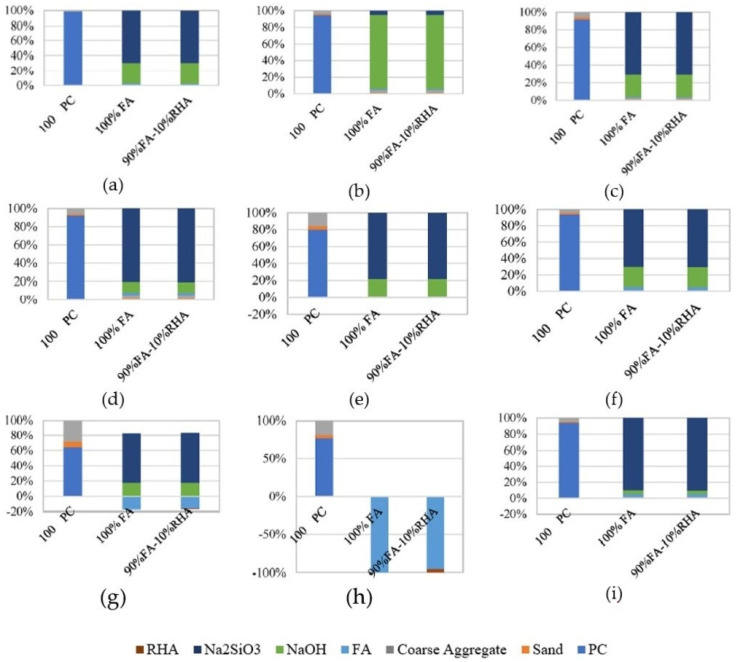
Contribution of the concrete ingredients to the following impact categories: (**a**) GWP—global warming potential, (**b**) AP—acidification potential, (**c**) EP—eutrophication potential, (**d**) POCP—photochemical ozone creation potential, (**e**) HTP—human toxicity potential, (**f**) ODP—ozone depletion, (**g**) FEW—freshwater ecotoxicity, (**h**) MWE—marine water ecotoxicity, and (**i**) AD—abiotic depletion [60].

Compared to OPC, the values for GHG emissions were lower in the MK-based geopolymer considering cradle-to-gate LCA. However, the opposite was noted with respect to the energy demand. The energy demand results were highly affected by the 3000 MJ of electricity needed to produce one ton of SS [85]. Abdulkareem et al. [148] studied the environmental performance of AAMs for a cradle-to-gate system boundary based on three different types of AAs. The normalized study findings demonstrated that for mortars of comparable strength, an AAM made with a two-part aqueous alkali solution (an SS solution and SH) and SH had a lower environmental impact than OPC mortar, while an AAM activated with SS powder had a higher impact. The importance of AAs was identified in the contribution analysis. High energy consumption in the production of SS powder leads to a sufficient environmental burden, while the best environmental performance corresponded to the AAM made with SH. When solely GWP was investigated, all the AAMs had more favourable results than OPC [148]. A study conducted by Fernando et al. [59] that evaluated an FA geopolymer, a blended AAC mixture in which 10% of FA was substituted with RHA, and OPC concrete found that the AA (a combination of commercially available SS and SH) was responsible for around 74% of the GHG emissions results. For reference to the LCA of parameters other than GWP, the impact categories considered are shown in Figure 7. When the normalised results were investigated, a higher impact of the AAC mixtures was reported for all categories except for MWE. For all eight impact categories (excluding MWE), AAs contributed approximately 80–97% of the values in the different categories [60].

While using industrial residue as an AA may not decrease the total GWP of AAMs, it might result in lower toxicity (in terms of terrestrial, freshwater, and marine environments) and less ozone depletion. So, the least processed raw materials produce the best outcomes across all categories [28]. As opposed to SS solutions, the RHA-derived solution has been proven to improve the environmental sustainability of AABs [114]. AAs derived from SH solutions and two different soluble silicate solutions, namely, commercial SS and chemically modified RHA, were investigated by Passuello et al. [118]. It was demonstrated that the selection of the activator impacted many categories evaluated in the LCA of the pastes. On the other hand, AABs produced using RHA resulted in lower impacts than other evaluated geopolymer pastes for all the categories considered [118]. Mellado et al. [67] investigated the carbon footprint of geopolymeric mortars utilising an alternative AA by refluxing RHA in an SH solution. It was compared with OPC and a conventional geopolymeric mortar (AA solution of commercial waterglass and SH). Consequently, in the case of the geopolymeric mortar, the synthesis of commercial waterglass was determined to be the most significant contributor to CO_2_ emissions. The substitution of the conventional waterglass with the RHA-derived waterglass led to a 50% reduction in emissions related to binders. When compared with OPC mortar, this value was 63% [67].

Moreover, generating a WGP-based alternative AA resulted in 18% lower CO_2_ emissions than the generation of SS [101]. Abdulkareem et al. [8] performed a comparative LCA in which AAMs produced using chemically modified one- and two-part waste-derived activators were compared to conventionally used ones. For this purpose, they used WGP and RHA. In the findings, it was determined that the AAMs with WGP and RHA resulted in 62%, 61%, 56%, and 76% lower values in the four investigated impact categories (climate change, fossil depletion, photochemical ozone creation, and terrestrial acidification) compared to the conventional activators [2]. On the other hand, Samarakoon et al. investigated WGP’s utilisation as a partial activator with SH. An 84% CO_2_ emissions reduction value compared to the OPC mixture was noted when WGP was part of the activator [146]. In the case of AAC, a conventional activator (SS consisting of SH and a commercial waterglass) could contribute up to 50% of the CO_2_ emissions. However, when an alternative activator containing SH and olivine nano-silica was used, this value was reduced to 41% [59]. Overall, it can be concluded that beneficial results can be achieved if conventional AAs are substituted by ones containing either industrial or agricultural waste.

### 3.3. Regional Context

The studies considered in this review were based on scenarios in the following regions: Australia [10,47,144,146,147], Brazil [118], the Czech Republic [130], the European Union (EU) [8,59,67,130,142], India [28], Malaysia [145], South Korea [4], Spain [67], Sri Lanka [60], the United States of America (USA) [85], and Vietnam [114]. Although concerning different countries, other studies did not touch upon the regional context. McLellan et al. [10] examined the carbon impacts of OPC and geopolymer pastes based on Australian practices. The assessment covered the life cycle stages up to the production of the binder. It was recognised that the key sources of geopolymers’ environmental “costs” are the source location, energy source, and transportation mode. Geopolymers resulted in significantly more favourable environmental impacts when only production was considered. However, a negative impact was identified for long distances when transportation was included in the assessment. Hence, the impact of transportation was higher in the geopolymer than in the OPC concrete. It was concluded that the geopolymers whose raw materials could be gathered from short distances could result in up to 64% lower GHG emissions than OPC. Through optimising transport, further potential emissions reductions were identified. Overall, it was concluded that the application of geopolymers should be assessed with respect to the specific location since this is one of the most determinant aspects [10]. Another study based on Australian practices investigated the utilisation of FA geopolymers. The authors found that emissions results for geopolymer are largely affected by the low local availability and larger distances for FA compared to OPC [147].

Mellado et al. [67] investigated AAMs based on data for Spain, and one section of the study examined emissions associated with the components of the binder, excluding mixing and curing but including the external grinding of fluid catalytic cracking catalyst (FCC) and RHA as well as the transportation of the materials from their point of origin. The contribution of transportation for OPC was 2%, while for AAMs this value was equal to 14% when the binder was made with FCC, commercial waterglass, SH, and water. In the case of replacing commercial waterglass with RHA, the contribution of transportation was 15%. This was also mainly due to the more considerable distances associated with the raw materials for AAMs [67]. Another study examined the environmental impact assessment of an AAC from cradle to pre-construction based on South Korean data. The findings suggested that, unlike OPC concrete, aggregate transportation substantially impacted the AAC [4]. An environmental investigation of an FA geopolymer and blended AAC with an FA/RHA ratio of 90/10 was undertaken in the Sri Lankan context. It was established that only 7–8% of the overall emissions from OPC concrete and AACs were attributable to transportation [60].

**Figure 8:**
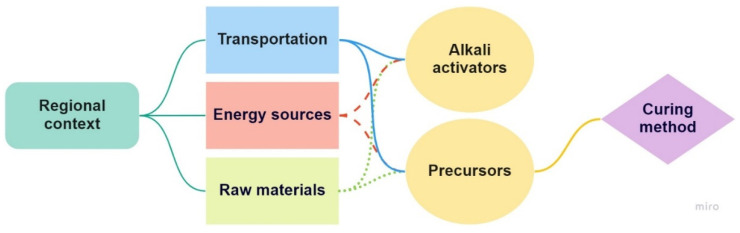
Interconnectivity of the major contributors to the environmental performance of AABs.

Since one of the most influential factors in the LCA of alternative binders is transportation, Nguyen et al. [85] performed an LCA for five binder types, namely, OPC, blended OPC with slag (SC), blended OPC with FA (FAC), an MK-based geopolymer (MKG), and high-volume limestone alkali-activated slag cement (HLAASC), focusing on transportation. The assessment was based on US practices and covered the binder’s cradle-to-gate system boundary. It was concluded that all the studied alternatives reduce GHG emissions. HLAASC reduced GHG emissions and energy consumption by as much as 95% and 83%, respectively, while FAC and SC effected comparable decreases in both GHG emissions and energy consumption. MKG decreased GHG emissions compared to OPC, SC, and FAC but not energy consumption. In the case of blended cements, the contribution of transportation was less than 5%, whereas it amounted to about 12% for MKG and 46% for HLAASC. Moreover, transportation was a critical contributor to the GHG emissions of all the investigated types of binders by up to 80% [85]. The overall interconnectivity of the main factors influencing the environmental performance of AABs is presented in Figure 8.

## 4. Obstacles Related to Economic Performance

**Figure 9:**
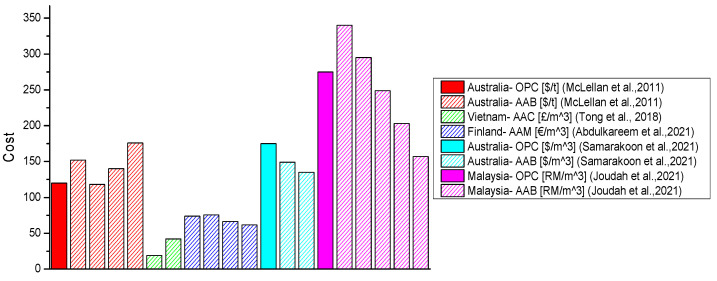
Economic performance of alkali-activated materials [8,10,114,145,146].

Economic performance is the least-studied aspect and, if investigated, has usually been conducted with environmental performance studies. According to Figure 9, we cannot generate straightforward conclusions regarding the economic performance of AAMs. This shows the complexity of the issue. Similar to LCA, the specification of the system boundary and the functional unit for LCC varied between studies (e.g., either m^3^ or t). In addition, it is difficult to compare the results because of the various currencies used depending on the region in which the studies were performed. Hence, identifying the main factors impacting such cost data of AAMs is essential. Key challenges regarding LCC include expensive feedstocks and transportation costs connected to their acquisition. Benefits could be generated by carefully choosing the sources of raw materials [10]. Hence, the local availability of primary and secondary raw materials will positively impact the total cost of AAMs [8]. Nguyen et al. investigated costs for alternative cementitious binders such as OPC, SC, FAC, MKG, and HLAASC (check Section 3.3 for a detailed explanation of mixes). It was shown that all alternatives to OPC presented environmental advantages, but none of them could overcome the cost obstacle [85]. However, including LCC in future studies on AABs is essential since their application in the related industry would be hindered by their high cost. Optimisation should be achieved by considering the critical challenging factors of certain mixes and addressing them to generate a competitively priced product without compromising its technical and environmental performance.

### 4.1. Precursors

The contribution of the precursor type used to an AAM’s cost is based on its production cost, the distance for which it must be transported, and the type of curing method employed (i.e., heat/ambient curing). Samarakoon et al. found that when WGP was used as both the partial activator and precursor, a 15% decrease in cost compared to an OPC binder was achieved [146]. Joudah et al. [145] investigated the production cost of AAMs made with FA, GGBFS, and WCeP. About fivefold higher values were obtained for WCeP compared to FA in terms of the cost for preparation as a raw material. The level of energy consumption for producing GGBFS was significantly higher than WCeP and FA. Additionally, GGBFS was associated with the highest transportation distance from all the precursor ingredients. Hence, the production costs of GGBFS, WCeP, and FA were RM 449 per t, RM 169 per t, and RM 34 per t (RM—Ringgit Malaysia), respectively. The 40% replacement of GGBFS with FA decreased costs by 54% and 43% compared to AAMs without FA and OPC mortar, respectively. A lower cost than OPC mortar was found for AAMs with a replacement ratio of GGBFS with FA starting from 20% [145]. Moreover, Fernando et al. compared the cost of concrete with FA as a precursor and a mix of FA and RHA in a study on AAC. It was shown that an increase in the final cost of a product due to adding RHA to the precursor was about 3% [60]. We can conclude that the production process and transportation distance of the precursor’s raw materials play a major role in the cost analysis of AABs.

### 4.2. Alkali Activators

In most of the studies concerning the LCC of AAMs, the type of AA used has been recognised as one of the most influential factors [8,28,60,85,114,146]. Regarding the cradle-to-gate LCC of MK-based geopolymers, its cost was higher than that of OPC. SS was identified as one of the main contributors [85]. Moreover, in a study by Fernando et al., a 48% increment was observed for AACs compared to OPC concrete, which was mainly affected by the high cost of AAs. In this study, AAs accounted for around 74% of the total cost of AAC [60]. On the other hand, in the studies that focused on alternative activators, a decrease in the total cost of AABs was visible [8,28,114,146]. Abdulkareem et al. identified that by producing waste-derived AAMs (i.e., with alternative activators, such as WGP and RHA), a range of 9–19% cost savings could be achieved compared to the conventional counterpart [8]. When AAMs were compared to OPC, the use of WGP as a partial activator could lead to a 23% reduction in cost [146]. Tong et al. showed that RHA-derived waterglass could lead to an almost 55% reduction in cost for the activation of AAC. Based on volume, these savings would be equal to about 22% per m^3^ [114]. Moreover, in a cost analysis on six different AAMs, 14% savings were achieved compared to OPC mortar, where a combination of RM and desulphurisation gypsum (DG) was used as an activator [28]. Using alternative sources was found to generate positive outcomes, even though AAs are among the primary causes of the high costs of AAMs.

### 4.3. Regional Context

The transportation costs for the raw materials used in AABs are especially important for relatively isolated regions such as Australia. McLellan et al. [10] examined the LCC of OPC and geopolymer pastes based on Australian practices. The assessment covered the life-cycle stages up to the production of the binder. It was recognised that the critical factors influencing the costs of geopolymers are the source location, the energy source, and the transportation mode. When only production was considered, geopolymers were acknowledged as potentially competitive in terms of cost. However, when transportation was included in the assessment, a negative impact was observed due to long distances. Moreover, the influence of transport on cost was more significant for the geopolymer paste than the OPC paste. Compared to OPC, the difference ranged from a 7% reduction to a 39% increase for the geopolymer paste. Through optimising transport, further cost reductions could be afforded [10]. On the other hand, in a study based on US data, the use of MK-based AAB resulted in a fivefold higher cost than OPC. The scarcity of the mineral in the USA and supply-related costs were recognised as the main contributors to such a trend. Therefore, transportation was a key contributor to the cost for all investigated types of binders, corresponding to increases of up to 65% [85]. The overall interconnectivity of the main contributors to AABs’ economic performance could also be related to the diagram shown in Figure 8. Hence, the utilisation of locally available raw materials for producing AABs would also lead to lower costs.

## 5. Conclusions

The investigation of alternative materials’ technical performance and economic and environmental benefits has been acknowledged as a crucial factor for the broader use of such materials in the building industry. However, there is still a lack of studies covering these three pillars while developing novel material solutions. Since alkali-activated binders (AABs) have been identified as a sustainable alternative in the cement industry for decades, this study aimed to validate such claims and identify the main obstacles to their broader application. The findings of this state-of-the-art review can benefit enthusiasts working on the development of AABs by identifying key concerns for the application of their products on a large scale. The adoption of green materials could be more efficiently accelerated if laboratory-scale projects were based on a robust approach.

Based on this literature review, the following conclusions could be drawn:

- The chemical compositions of the precursor and alkali activator used highly affect the material properties of AABs. There is still a lack of studies covering a wide range of sources for alternative precursors and alkali activators, which has led to the limited application of such AABs.
- Concentrating investigations on locally available raw materials seems to be one of the most feasible solutions for adopting AABs on a larger scale in the industry. Future studies should focus on developing tools to identify the main factors influencing the variability of AABs’ material properties based on raw materials (e.g., machine learning).
- The interconnectivity between the most influential factors regarding AABs’ environmental and economic performance (i.e., the regional context, transportation, energy sources, raw materials, alkali activators, precursors, and the curing method employed) demonstrates the importance of the consistent definition of both functional units and system boundaries. Evaluating only part of the critical factors can lead to results that would not realistically represent the situation when the product is applied in the industry.
- The environmental benefits of utilizing feedstocks from construction and demolition waste could substantially improve sustainable pathways in the construction industry by focusing on circularity. Hence, alkali-activated materials employing these raw materials should be the focus of future research.
- Studies on the economic performance of AABs are the rarest of the three pillars considered herein. Expensive feedstocks constitute a critical challenge for the economic performance of AABs, which is usually connected with high transportation costs. It has been demonstrated that by using a thorough approach, the cost of AABs can be decreased without compromising the other two pillars (i.e., technical and environmental performance).
- Future research may benefit from using the multi-criteria analysis of alkali-activated materials to assist the development of comprehensive methodologies. Overall, optimisation should be reached by considering the most challenging factors in producing alkali-activated materials, thereby achieving favourable trade-offs between technical, environmental, and economic performance.

## Figures and Tables

**Table 2:** Chemical compositions of alternative precursors.

[m%]	Al_2_O_3_	CaO	Fe_2_O_3_	K_2_O	MgO	Na_2_O	SO_3_	SiO_2_
RM	21.7 ± 6.5	7.9 ± 7.1	30.2 ± 15.3	0.4 ± 0.4	0.5 ± 0.7	5.3 ± 4.7	0.4 ± 0.5	13.9 ± 7.0
RHA	0.4 ± 0.3	1.2 ± 1.2	0.5 ± 0.7	1.7 ± 1	0.6 ± 0.4	0.1 ± 0.1	0.2 ± 0.3	91.6 ± 3.2
WBP	16.8 ± 7.1	12.2 ± 7.8	7.1 ± 3.7	5.6 ± 8.7	2.8 ± 2.4	0.7 ± 0.6	0.9 ± 1.1	54.5 ± 11.3
WGP	8.3 ± 8	8.7 ± 4.5	0.8 ± 1.6	1.9 ± 2.0	1.0 ± 0.9	9.2 ± 5.2	1.1 ± 2.7	61.7 ± 10.3

**Table 3:** Comparison between data regarding environmental impact assessments from the literature.

Source	Functional Unit	System Boundary	Transportation		Heat Curing		LCA Method	EI Categories Except GWP	
			Yes	No	Yes	No		Yes	No
[5]	t	cradle-to-gate	N/S			x	-	x	
[6]	t	cradle-to-gate	N/S			x	-		x
[142]	t	cradle-to-gate		x	x		CML	x	
[10]	t	cradle-to-gate ^1^	x			x	-	x	
[4]	m^3^	cradle-to-preconstruction	x		x		-		x
[144]	m^3^	cradle-to-site	x		x		-		x
[67]	t	cradle-to-gate	x			x	-		x
[118]	t	cradle-to-gate ^1^	x		x		CML	x	
[59]	m^3^	cradle-to-gate	x			x	-		x
[130]	t	cradle-to-gate		x		x	-	x	
[114]	t	cradle-to-gate		x	x		-	x	
[147]	m^3^ and m^2^	cradle-to-gate/site	x		x		-		x
[148]	m^3^	cradle-to-gate	x		x		CML	x	
[81]	m^3^	cradle-to-gate		x	x		-	x	
[145]	t	cradle-to-gate	x			x	-	x	
[127]	m^3^	cradle-to-gate	x			x	-		x
[8]	m^3^	cradle-to-gate		x		x	ReCiPe	x	
[60]	m^3^	cradle-to-gate/end of construction	x		x		-		x
[146]	m^3^	cradle-to-gate	x		x		-		x
[28]	m^3^	cradle-to-gate	x			x	ReCiPe	x	
[47]	t	cradle-to-gate		x		x	-	x	
[101,102]	t	cradle-to-gate	N/S			x	-		x
[46]	t	cradle-to-gate		x		x	-	x	
[48]	t	cradle-to-gate		x		x	-	x	

^1^ mixing data excluded.

## Data Availability

Not applicable.
